# Serum uric acid and the risk of hypertension in a Middle Eastern cohort: A 10-year prospective study discovering BMI-mediated associations and gender-specific vulnerabilities

**DOI:** 10.1097/MD.0000000000045285

**Published:** 2025-10-17

**Authors:** Mohammadtaghi Sarebanhassanabadi, Seyed Reza Mirjalili, Pedro Marques-Vidal, Seyedeh Mahdieh Namayandeh, Ali Mirshamsi

**Affiliations:** a Yazd Cardiovascular Research Center, Non-communicable Diseases Research Institute, Shahid Sadoughi University of Medical Sciences, Yazd, Iran; b Department of Internal Medicine, BH10-642, Lausanne, Switzerland.

**Keywords:** cohort study, hypertension, hyperuricemia, obesity, serum uric acid, sex differences

## Abstract

Hypertension (HTN), a modifiable cardiovascular risk factor, remains a global public health challenge. Serum uric acid (SUA) has been implicated in HTN pathogenesis, but the evidence is conflicting. This study investigated the association between SUA and 10-year HTN incidence, stratified by sex and body mass index (BMI), in a Middle Eastern cohort. A 10-year prospective cohort analysis using data from 828 adults (53.5% male) aged 20 to 74 years in Yazd, Iran, sourced from the Yazd Healthy Heart Project. Participants free of baseline HTN (defined as systolic blood pressure/diastolic blood pressure < 130/80 mm Hg, no antihypertensive use, and no prior diagnosis) were categorized into hyperuricemic (SUA > 5.5 mg/dL [males], >4.6 mg/dL [females]) and normouricemic groups. Incident HTN was defined as systolic/diastolic blood pressure ≥ 130/80 mm Hg, antihypertensive use, or medical diagnosis. Hyperuricemia was associated with a 59% higher HTN risk (odds ratio [OR] = 1.59, 95% confidence interval [CI]: 1.14–2.20), but this attenuated after BMI adjustment (OR = 1.16, 95% CI: 0.73–1.85). Stratified analyses revealed borderline associations in men (adjusted OR = 1.79, 95% CI: 1.01–3.19, *P* = .05) and normal-weight individuals (BMI < 25 kg/m²: OR = 2.32, 95% CI: 1.04–5.22), persisting across adjustment models. No associations were observed in the women or overweight/obese subgroups. Hyperuricemic individuals exhibited higher baseline BMI and dyslipidemia (*P* < .01). Elevated SUA independently predicts HTN risk in men and normal-weight individuals, suggesting context-dependent mechanisms. Adiposity mediates the population-level association, while SUA’s direct vascular effects may dominate in leaner subgroups.

## 1. Introduction

Hypertension (HTN) remains a leading modifiable risk factor for cardiovascular disease, contributing significantly to global morbidity and mortality.^[1]^ According to the World Health Organization, elevated blood pressure affects over 1.3 billion adults worldwide and is implicated in approximately 10.8 million deaths annually, primarily due to ischemic heart disease, stroke, and renal failure.^[1,2]^ The prevalence of HTN continues to rise in parallel with urbanization, sedentary lifestyles, and dietary shifts toward processed foods, factors that amplify metabolic risk profiles in diverse populations.^[3]^ Despite advancements in antihypertensive therapies, primary prevention remains critical to reducing this burden, necessitating the identification of modifiable predictors of HTN.

Serum uric acid (SUA), the end product of purine metabolism, is a potential contributor in the pathogenesis of HTN.^[4]^ Observational and experimental studies suggest that hyperuricemia may promote endothelial dysfunction, oxidative stress, and renal microvascular damage, thereby contributing to increased vascular resistance and blood pressure elevation.^[5]^ Previous prospective studies have yielded conflicting results, with some reporting SUA as an independent predictor of HTN^[6]^ and others attributing this association to confounding by shared metabolic risk factors, such as insulin resistance, obesity, or subclinical renal impairment.^[7]^ Furthermore, data from Middle Eastern populations, characterized by distinct genetic, dietary, and lifestyle profiles, are scarce, limiting the generalizability of existing findings.^[8]^

Long-term prospective studies are critical to clarify SUA’s role in development of HTN, especially in populations with high baseline metabolic risk.^[9]^ To date, few investigations have evaluated the decade-long relationship between SUA and HTN incidence while rigorously accounting for confounders.^[10]^ Resolving these uncertainties holds clinical relevance, as SUA-lowering interventions could offer dual benefits in populations prone to hyperuricemia and cardiovascular disease.^[11]^

This study investigated the association between baseline SUA levels and the 10-year incidence of HTN using data from a community-based prospective cohort. We hypothesized that elevated SUA levels predict the risk of HTN, independently from confounders such as age, sex, adiposity, and metabolic syndrome components. By addressing gaps in longitudinal evidence and refining the understanding of uric acid’s etiological role, this work aims to inform targeted prevention strategies in high-risk populations.

## 2. Methods

### 2.1. Study design and study subjects

This cohort study utilized data from the Yazd Healthy Heart Project (YHHP), a comprehensive research initiative focused on investigating cardiovascular and metabolic disorders among individuals aged 20 to 74 residing in the urban areas of Yazd city (the capital of Yazd province, located in central Iran). The study utilized a cluster-based random sampling design, with the target sample size calculated through standard statistical estimation methods. Recruitment followed a geographically stratified framework: Yazd city was divided into 100 clusters, from which 20 households per cluster were randomly chosen. Within each selected household, one adult aged 20 to 74 years was randomly enrolled, resulting in a cohort of 2000 participants balanced by sex (1000 men and 1000 women). Data collection was carried out by the Yazd Cardiovascular Research Center in 2 phases: an initial baseline survey in 2005 to 2006 and a follow-up assessment roughly 10 years later, in 2015 to 2016. Extensive information about the YHHP, along with findings from previous research based on this cohort, has already been published.^[12,13]^

### 2.2. Ethical consideration

This study was carried out in accordance with the ethical standards outlined in the Declaration of Helsinki. Ethical approval was granted by the Ethics Committee of Shahid Sadoughi University of Medical Sciences (Approval code: IR.SSU.REC.1403.062). All participants provided written informed consent prior to enrollment. Data collection procedures were designed to ensure complete anonymity, with no personally identifiable information recorded or stored.

### 2.3. Main outcome

Incident HTN was defined according to the 2017 American College of Cardiology/American Heart Association guidelines as a systolic blood pressure ≥ 130 mm Hg, a diastolic blood pressure ≥ 80 mm Hg, antihypertensive medication use, or physician diagnosis documented in medical records.^[14]^

### 2.4. Biochemical measurements

Following a 12-hour overnight fast, venous blood samples were collected from the antecubital vein. The samples were then centrifuged to isolate serum for biochemical analysis. SUA, fasting blood glucose, and lipid profile components, including triglycerides, total cholesterol, high-density lipoprotein cholesterol, and low-density lipoprotein cholesterol, were quantified at the Afshar Hospital medical laboratory in Yazd, Iran, using a BT 3000 biochemical autoanalyzer (Rome, Italy). All assessments were carried out by trained laboratory personnel, with instrument calibration routinely performed to ensure measurement accuracy and consistency.

### 2.5. Covariates

Trained interviewers collected data through face-to-face surveys, which included questions on age, educational attainment, physical activity, and smoking status. Educational levels were classified into 3 categories: primary, high school, and higher (academic) education. Physical activity levels were assessed using the International Physical Activity Questionnaire and participants were subsequently grouped into low, moderate, or high activity categories based on standardized International Physical Activity Questionnaire criteria.^[15]^ Smoking status was defined as current cigarette smoking (yes/no). Data on frequency, amount, and duration of smoking were not consistently available across the cohort; therefore, the analysis was limited to the binary classification of current smoker versus nonsmoker (encompassing never-smokers and former smokers). Blood pressure measurements were conducted in alignment with the YHHP cohort protocol using a digital automatic sphygmomanometer (Omron M6 Comfort, Osaka, Japan). Anthropometric assessments, including weight (kg), height (cm), waist circumference, hip circumference, waist-to-hip ratio, and body mass index (BMI), were performed according to standardized procedures outlined in the cohort methodology.

### 2.6. Included participants

**Figure 1.**
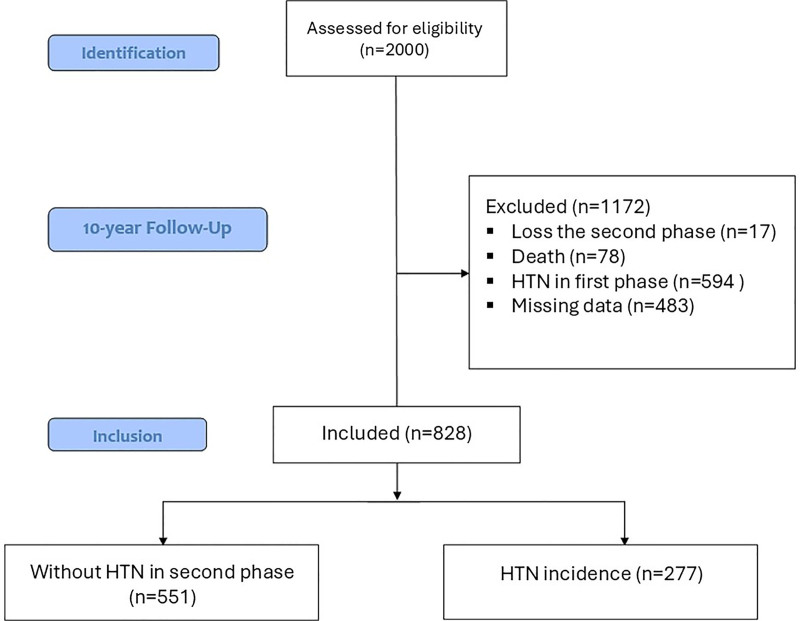
Participant flow diagram. Flowchart outlining the selection process of the Yazd Healthy Heart Project cohort. The final analysis included 828 participants, of whom 277 developed hypertension over the 10-year follow-up period.

The inclusion criteria for this analysis were: participation in the YHHP baseline exam, availability of complete baseline SUA and covariate data, and being free of HTN at baseline. Exclusion criteria included: prevalent HTN at baseline (n = 594), death during follow-up (n = 78), loss to follow-up (n = 17), or missing crucial data (n = 483). The primary reasons for missing data were loss to follow-up or incomplete laboratory records. We assumed the data to be missing at random after comparing available characteristics between those with and without missing data showed no significant differences. The study included the remaining 828 participants for the current research (Fig. 1).

### 2.7. Statistical analysis

Statistical analyses were performed using SPSS software, version 23 (SPSS Inc., Chicago, IL). Continuous variables were expressed as mean ± standard deviation, while categorical variables were summarized using frequencies and corresponding percentages.

Since there is no established consensus on SUA thresholds for HTN, these data-driven cutoffs were used instead of the conventional thresholds (≥7 mg/dL for males and ≥6 mg/dL for females), which are primarily used for diagnosing gout.^[16,17]^

A classification-based analysis was performed by dividing individuals into 2 groups based on their SUA levels: hyperuricemic and normouricemic. The threshold levels for hyperuricemia were determined using receiver operating characteristic curve analysis, selecting the cutoff values that maximized the Youden Index (SUA > 5.5 mg/dL for males and >4.6 mg/dL for females), ensuring optimal sensitivity and specificity for predicting HTN in this cohort. This approach was chosen over conventional gout thresholds to better reflect the association with cardiovascular risk in our population.

A one-way analysis of variance was conducted to evaluate the significant differences in the continuous variables between the hyperuricemic and normouricemic groups. In addition, logistic regression analysis was performed to assess the association between hyperuricemia and the likelihood of developing new-onset HTN, calculating odds ratios (ORs) with 95% confidence intervals (CIs). We acknowledge that for common outcomes like HTN (incidence rate of 33.5% in our cohort), the OR may overestimate the relative risk. However, logistic regression was selected for its stability in multivariate modeling compared to log-binomial regression. Three models were assessed, adjusting for relevant confounders. Odds ratios (ORs) with 95% CIs were reported. *Model I* was adjusted for age and sex; *Model II* included adjustments for age, sex, smoking, and physical activity; and *Model III* expanded upon Model II by also adjusting for systolic blood pressure, diastolic blood pressure, and BMI. A 2 -sided *P*-value of <.05 was deemed statistically significant for all analyses.

To assess the potential impact of participant exclusions, we first compared the baseline characteristics of included versus excluded participants (Table 1). Independent samples *t* tests and Chi-square tests were used for continuous and categorical variables, respectively. In addition, we conducted sensitivity analyses using multiple imputation for missing data (5 imputed datasets using fully conditional specification). Logistic regression models for incident HTN were repeated in the imputed sample, and effect estimates were compared with those from the complete-case analysis.

**Table 1:** Baseline clinical characteristics and biological variables of the participants.

	Included	Excluded	*P*-value
Number of participants	828	1172	
Age (yr)	42.26 ± 13.01	53.34 ± 15.00	<.001
Male (%)	443 (53.5)	555 (47.4)	.007
Education (%)			<.001
Primary	370 (46.8)	817 (70.4)	
High school	322 (40.7)	265 (22.8)	
Academic	99 (12.5)	79 (6.8)	
Anthropometry			
Waist circumference (cm)	91.14 ± 11.88	95.17 ± 12.21	<.001
Body mass index (kg/m^2^)	25.62 ± 4.27	26.49 ± 4.58	<.001
Current smokers (%)	145 (17.5)	204 (17.6)	.97
Physical activity (%)			<.001
Low	345 (63.1)	598 (72.6)	
Moderate	172 (31.4)	193 (23.4)	
Vigorous	30 (5.5)	33 (4)	
Blood pressure (mm Hg)			
Systolic	119.75 ± 9.07	134.12 ± 16.59	<.001
Diastolic	78.37 ± 5.38	85.46 ± 9.63	<.001
Diabetes (%)	109 (14.7)	136 (37)	<.001
Blood levels (mg/dL)			
Fasting glucose	95.71 ± 37.81	107.96 ± 49.80	<.001
Total cholesterol	191.25 ± 43.54	203.17 ± 44.71	<.001
LDL-cholesterol	104.00 ± 36.29	111.78 ± 36.22	<.001
HDL-cholesterol	53.96 ± 13.56	53.91 ± 13.76	.93
Triglycerides	160.75 ± 101.48	185.90 ± 111.14	<.001

Results are expressed as the number of participants (column percentage) for categorical variables and as average ± standard deviation for continuous variables. Between group comparisons performed using Chi-square test for categorical variables and by the Student *t* test for continuous variables.

HDL-C = high-density lipoprotein cholesterol; LDL-cholesterol = low-density lipoprotein cholesterol.

## 3. Results

This study analyzed a cohort of 828 individuals, comprising 53.5% males, over an average follow-up span of 10 years. The baseline clinical characteristics and biological variables of the participants, categorized by SUA levels, are detailed in Table 2. Notably, hyperuricemic individuals exhibited an elevated BMI and a greater prevalence of dyslipidemia, factors closely associated with the onset and progression of HTN.

**Table 2:** Baseline clinical characteristics and biological variables of the participants according to serum uric acid levels.

	Normouricemic	Hyperuricemic	*P*-value
Number of participants	628	200	
Age (yr)	42.01 ± 12.97	43.05 ± 13.17	.33
Gender (%)			.10
Male	326 (51.9)	117 (58.5)	
Female	302 (48.1)	83 (41.5)	
Education (%)			.91
Primary	284 (46.9)	86 (46.2)	
High school	244 (40.3)	78 (41.9)	
Academic	77 (12.7)	22 (11.8)	
Anthropometry			
Weight/hip ratio	0.88 ± 0.10	0.91 ± 0.08	<.001
Body mass index (kg/m^2^)	25.00 ± 4.13	27.55 ± 4.14	<.001
Current smokers (%)	111 (17.7)	34 (17.0)	.83
Physical activity (%)			.56
Low	268 (64.0)	77 (60.2)	
Moderate	127 (30.3)	45 (35.2)	
Vigorous	24 (5.7)	6 (4.7)	
Blood pressure (mm Hg)			
Systolic	119.31 ± 9.12	121.12 ± 8.77	.01
Diastolic	78.22 ± 5.46	78.86 ± 5.11	.14
Diabetes (%)	70 (11.1)	16 (8.0)	.20
Blood levels (mg/dL)			
Fasting glucose	96.06 ± 40.53	94.58 ± 27.64	.56
Total cholesterol	186.22 ± 41.49	207.08 ± 46.07	<.001
LDL-cholesterol	100.96 ± 34.46	113.73 ± 40.18	<.001
HDL-cholesterol	54.30 ± 13.28	52.90 ± 14.37	.20
Triglycerides	148.24 ± 92.58	200.03 ± 117.25	<.001

Results are expressed as the number of participants (column percentage) for categorical variables and as average ± standard deviation or median (interquartile range) for continuous variables. Between group comparisons performed using Chi-square for categorical variables and analysis of variance or nonparametric tests for continuous variables.

HDL-C = high-density lipoprotein cholesterol, LDL-cholesterol = low-density lipoprotein cholesterol.

Table 3 illustrates the association between SUA levels and the risk of HTN in the overall study population. HTN incidence was reported in 277 (33.5%) of the participants. It should be noted that due to this high incidence rate, the odds ratios reported hereafter may overestimate the relative risk. Univariate analysis revealed that hyperuricemia was linked to a significantly elevated risk of HTN (OR = 1.59, 95% CI: 1.14–2.20). This relationship remained statistically robust even after adjusting for potential confounders in Model II. However, upon further adjustment in Model III, which incorporated BMI as an additional confounding factor, the association lost statistical significance (OR = 1.16, 95% CI: 0.73–1.85), suggesting that BMI may mediate this relationship.

**Table 3:** Risk of hypertension according to serum uric acid levels stratified by gender.

	Normouricemic	Hyperuricemic	*P*-value
All participants			
Crude	1	1.587 (1.143–2.204)	.006
Model I	1	1.590 (1.121–2.256)	.009
Model II	1	1.578 (1.018–2.445)	.042
Model III	1	1.159 (0.726–1.850)	.537
Men			
Crude	1	1.540 (0.993–2.387)	.054
Model I	1	1.873 (1.163–3.018)	.010
Model II	1	1.759 (1.007–3.073)	.047
Model III	1	1.786 (1.01–3.193)	.050
Women			
Crude	1	1.669 (1.015–2.745)	.044
Model I	1	1.351 (0.799–2.286)	.262
Model II	1	1.422 (0.686–2.951)	.344
Model III	1	1.327 (0.619–2.844)	.468

*Note*: Due to the high incidence of hypertension (33.5%), the reported odds ratios may overestimate the relative risk.

Results are expressed as odds ratio and (95% confidence interval). Model I: adjusted for age; Model II: adjusted for age, smoking, and physical activity; Model III: adjusted for age, smoking, physical activity, BMI, SBP, and DBP.

BMI = body mass index, DBP = diastolic blood pressure, SBP = systolic blood pressure.

As shown in Table 3, the gender-stratified analysis identified a borderline association between hyperuricemia and the risk of HTN in men. However, in women, a notable association emerged only in the unadjusted model, but this association diminished and lost statistical significance after adjustments for confounding factors.

Based on the mediating role of BMI, the participants were divided into 2 groups: normal weight (BMI 18.5–24.9) and overweight/obese (BMI ≥ 25). A BMI-stratified analysis was conducted to examine the association between hyperuricemia and HTN. In the normal weight group, a significant association between hyperuricemia and HTN was observed, which remained robust even after adjusting for confounding factors. However, this association was absent in the overweight and obese groups. The results are presented in Table 4.

**Table 4:** Risk of hypertension according to serum uric acid levels stratified by body mass index classification.

All participants	Normouricemic	Hyperuricemic	*P*-value
Normal weight			
Crude	1	2.104 (1.169–3.785)	.013
Model I	1	2.346 (1.251–4.401)	.008
Model II	1	2.652 (1.211–5.809)	.015
Model III	1	2.323 (1.035–5.215)	.041
Overweight and obese			
Crude	1	1.146 (0.765–1.716)	.509
Model I	1	1.125 (0.733–1.727)	.589
Model II	1	1.027 (0.599–1.761)	.923
Model III	1	1.081 (0.619–1.887)	.785

*Note*: Due to the high incidence of hypertension (33.5%), the reported odds ratios may overestimate the relative risk.

Results are expressed as odds ratio and (95% confidence interval). Model I: adjusted for age and sex; Model II: adjusted for age, sex, smoking, and physical activity; Model III: adjusted for age, sex, smoking, physical activity, SBP, and DBP.

DBP = diastolic blood pressure, SBP = systolic blood pressure.

We then performed multiple imputation for missing covariates (5 imputations using chained equations) and repeated the logistic regression analyses. Results were materially unchanged compared with the complete-case analysis. Specifically, hyperuricemia remained associated with an increased risk of incident HTN in men (imputed adjusted OR = 1.82, 95% CI: 1.05–3.20) and in normal-weight participants (imputed adjusted OR = 2.28, 95% CI: 1.02–5.10). No significant associations were observed in women (imputed adjusted OR = 1.29, 95% CI: 0.61–2.72) or overweight/obese participants (imputed adjusted OR = 1.09, 95% CI: 0.63–1.86). These findings support the robustness of our conclusions and suggest that selection bias from exclusions is unlikely to explain the observed associations.

## 4. Discussion

This 10-year prospective cohort study offers significant insights into the interplay between SUA levels and HTN risk, highlighting distinct associations attributed to sex and adiposity. Initially, hyperuricemia was associated with 59% increased odds of HTN in the overall cohort. However, this association lost significance after adjusting for BMI, underscoring the critical role of obesity in this relationship. The findings align with previous research indicating that body fat confounded the relationship between SUA and HTN through shared pathways such as insulin resistance and renal dysfunction.^[18]^ Stratified analyses indicated subgroup-specific risks: hyperuricemia was an independent predictor of HTN in men (OR = 1.79, 95% CI: 1.01–3.19, *P* = .05) and in normal-weight individuals (OR = 2.32, 95% CI: 1.04–5.22), implying that the vascular toxicity of SUA may be more significant in these groups.

The sex-specific disparities may stem from biological and hormonal differences. Estrogen’s uricosuric effects in premenopausal women likely reduce SUA retention, mitigating its vascular toxicity.^[19]^ Conversely, testosterone in men may amplify SUA-induced oxidative stress and endothelial dysfunction, exacerbating renal sodium retention and blood pressure elevation.^[20,21]^ Furthermore, visceral adiposity, which is more common in men,^[22]^ may interact with SUA to trigger inflammatory pathways (such as the NLRP3 inflammasome) and reduce nitric oxide bioavailability, thereby increasing the risk of HTN.^[23–25]^

In individuals of normal weight, the direct pathological mechanisms of SUA may be predominant. Experimental models indicate that SUA inhibits endothelial nitric oxide synthase, enhances reactive oxygen species production, and activates the intrarenal renin–angiotensin–aldosterone system independent of adiposity.^[26]^ Individuals with a lean physique may be deficient in the compensatory mechanisms found in obesity, such as the anti-inflammatory effects of adiponectin, which may increase their susceptibility to the vascular toxicity associated with SUA.^[8,27]^ The metabolic overload associated with obesity, characterized by hyperinsulinemia and leptin resistance likely diminishes the role of SUA, positioning it as a secondary factor in the pathogenesis of HTN.^[4,28,29]^

This study’s strengths lie in its prospective design, thorough adjustment for confounders, and emphasis on an understudied Middle Eastern cohort, which enhances the generalizability to populations at high metabolic risk. Nevertheless, the limitations warrant attention. The small hyperuricemic subgroup and the borderline significance observed in men (*P*-value = .05) require careful interpretation. Residual confounding due to unmeasured variables, such as dietary purines^[30]^ and genetic polymorphisms,^[31]^ along with the observational design, limits the ability to draw causal inferences. Furthermore, the use of sex-specific SUA cutoffs optimized for HTN prediction via receiver operating characteristic analysis, rather than conventional gout thresholds, may limit direct comparability with other studies. While this approach enhanced the predictive model for our cohort, future studies should validate these thresholds externally and compare them against standard definitions. Another limitation of our study is the relatively high proportion of excluded participants, mainly due to prevalent baseline HTN and missing data. Excluded individuals were generally older, with higher cardiometabolic risk, which could lead to selection bias. However, our sensitivity analyses using multiple imputation yielded results consistent with the complete-case analyses, suggesting that the observed associations are robust. Nonetheless, the possibility of residual selection bias cannot be entirely ruled out, and caution is warranted when generalizing our findings to broader populations. An additional limitation is the unavailability of hemoglobin A1c data at baseline. While we defined diabetes status using fasting blood sugar and physician diagnosis, the absence of hemoglobin A1c, a marker of long-term glycemic control, means we could not fully account for the potential confounding influence of subclinical or poorly controlled dysglycemia on the observed associations.

Clinically, these findings advocate for targeted SUA monitoring in men and normal-weight individuals, even at sub-gout thresholds, to identify high-risk subgroups for early intervention. Lifestyle modifications, such as reducing fructose intake and promoting physical activity, could mitigate SUA-related risks while addressing broader metabolic health.^[32]^ Future research should prioritize mechanistic studies to clarify sex- and BMI-specific pathways, alongside randomized trials testing SUA-lowering therapies (e.g., allopurinol) for HTN prevention in vulnerable subgroups.^[33]^

## 5. Conclusion

In conclusion, SUA emerged as an independent risk factor for the development of HTN in men and normal-weight individuals, highlighting the importance of subgroup-specific approaches. These findings underscore the value of integrating metabolic and demographic heterogeneity into public health initiatives, paving the way for precision-based interventions to curb the global burden of HTN.

## Acknowledgments

The authors would like to acknowledge the invaluable contributions of the participants and colleagues involved in the Yazd Healthy Heart Project, as well as the support provided by Shahid Sadoughi University of Medical Sciences in facilitating this research endeavor.

## Author contributions

**Conceptualization:** Mohammadtaghi Sarebanhassanabadi, Seyedeh Mahdieh Namayandeh, Ali Mirshamsi.

**Data curation:** Seyed Reza Mirjalili, Pedro Marques-Vidal, Seyedeh Mahdieh Namayandeh, Ali Mirshamsi.

**Formal analysis:** Mohammadtaghi Sarebanhassanabadi, Pedro Marques-Vidal.

**Investigation:** Mohammadtaghi Sarebanhassanabadi, Seyed Reza Mirjalili, Seyedeh Mahdieh Namayandeh.

**Methodology:** Mohammadtaghi Sarebanhassanabadi, Seyed Reza Mirjalili, Pedro Marques-Vidal, Ali Mirshamsi.

**Resources:** Mohammadtaghi Sarebanhassanabadi, Seyedeh Mahdieh Namayandeh.

**Software:** Seyed Reza Mirjalili, Ali Mirshamsi.

**Supervision:** Pedro Marques-Vidal, Ali Mirshamsi.

**Validation:** Mohammadtaghi Sarebanhassanabadi, Seyed Reza Mirjalili, Pedro Marques-Vidal, Seyedeh Mahdieh Namayandeh.

**Writing – original draft:** Mohammadtaghi Sarebanhassanabadi, Seyed Reza Mirjalili, Ali Mirshamsi.

**Writing – review & editing:** Pedro Marques-Vidal, Ali Mirshamsi.
